# Supplementary material for: Trends and factors of botanical dietary supplement use among US adults with COPD from 1999 to 2016
Source: PLoS One. 2020 Sep 29;15(9):e0239674. doi: 10.1371/journal.pone.0239674 (PMC7523968; doi:10.1371/journal.pone.0239674)
Supplement: Attachment — Submitted filename: Response to Reviewers.docx [file pone.0239674.s001.docx]

Response to Reviewers

| **EDITOR'S SPECIFIC COMMENTS:** | **Revise** |
| --- | --- |
| 1. Please ensure that your manuscript meets PLOS ONE's style requirements, including those for file naming. The PLOS ONE style templates can be found at  https://journals.plos.org/plosone/s/file?id=wjVg/PLOSOne_formatting_sample_main_body.pdf and  https://journals.plos.org/plosone/s/file?id=ba62/PLOSOne_formatting_sample_title_authors_affiliations.pdf | Thanks. We have made changes based on PLOS ONE’s style. |
| 2. We noticed minor instances of text overlap with the following previous publication(s), which need to be addressed:  (1) https://www.sciencedirect.com/science/article/abs/pii/S2213219817307419?via%3Dihub  (2) https://acsjournals.onlinelibrary.wiley.com/doi/full/10.1002/cncr.31183  The text that needs to be addressed involves the Results section (lines 190-195) and the Discussion section (lines 244-285).  In your revision please ensure you cite all your sources (including your own works), and quote or rephrase any duplicated text outside the methods section. Further consideration is dependent on these concerns being addressed. | Thanks. We have rewritten these two parts and cited qualified references. |
| **COMMENTS FROM REVIEWER #1:** | **Revise** |
| **1.**  It would be useful for the authors to note an example of the supplements being discussed | Thanks for this comment. We discussed one specific herb use in COPD management in the third paragraph of discussion. |
| **2.**  The authors have to be very careful in this cross sectional analysis to NOT imply causality ( i.e.- supplement users had fewer hospitalizations and less anemia, but you can't say anything about directionality of this association) | Thank you for this comment. We have tried our best to avoid such statements, and in limitation part, we have discussed such causality issues of cross-sectional study. |
| **3.**  SES factors were the strongest predictors of use ( and these are also strong predictors of health outcomes in the US) - thus- what was seen might still be a function of unmeasured SES confounders ( even in the multivariate analysis) | Thank you for pointing out SES factors. Yes, you are right, SES factors may be the strongest predictors of BDS use. However, due to the limitations of NHANSE data, not all SES factors can be observed or controlled. We have discussed more about this issue in the discussion and limitation parts. |
